# Prospective minimally invasive pancreatic resections from the IGOMIPS registry: a snapshot of daily practice in Italy on 1191 between 2019 and 2022

**DOI:** 10.1007/s13304-023-01592-7

**Published:** 2023-07-20

**Authors:** Ugo Boggi, Greta Donisi, Niccolò Napoli, Stefano Partelli, Alessandro Esposito, Giovanni Ferrari, Giovanni Butturini, Luca Morelli, Mohammad Abu Hilal, Massimo Viola, Fabrizio Di Benedetto, Roberto Troisi, Marco Vivarelli, Elio Jovine, Alessandro Ferrero, Umberto Bracale, Sergio Alfieri, Riccardo Casadei, Giorgio Ercolani, Luca Moraldi, Carlo Molino, Raffaele Dalla Valle, Giuseppe Ettorre, Riccardo Memeo, Giacomo Zanus, Andrea Belli, Salvatore Gruttadauria, Alberto Brolese, Andrea Coratti, Gianluca Garulli, Renato Romagnoli, Marco Massani, Felice Borghi, Giulio Belli, Roberto Coppola, Massimo Falconi, Roberto Salvia, Alessandro Zerbi, the IGOMPIS registry, Emanuele F. Kauffmann, Giovanni Capretti, Luana Genova, Matteo De Pastena, Michele Mazzola, Alessandro Giardino, Matteo Palmieri, Alberto Manzoni, Vittoria Barbieri, Roberto Ballarin, Gianluca Rompianesi, Roberta Rossi, Laura Mastrangelo, Serena Langella, Mariangela Ilardi, Roberta Menghi, Claudio Ricci, Andrea Gardini, Donata Campra, Enrico Crolla, Sara Cecconi, Roberto L. Meniconi, Valentina Ferraro, Marco Brizzolari, Francesco Izzo, Davide Cintorino, Stefano Marcucci, Giuseppe Giuliani, Luigi Veneroni, Francesco Moro, Cristina Nistri, Damiano Caputo, Baiocchi Gianluca, Vincenzo Mazzaferro

**Affiliations:** 1 Division of General and Transplant Surgery, University of Pisa, Pisa, Italy; 2 Pancreatic Surgery Unit, Department of Biomedical Sciences, Humanitas University, Pieve Emanuele, Italy; 3 Pancreatic Surgery Unit, Pancreas Translational and Clinical Research Center, OSR ENETS Center of Excellence, IRCCS San Raffaele Scientific Institute, Milan, Italy; 4 General and Pancreatic Surgery Unit, Pancreas Institute, University of Verona, Verona, Italy; 5 Division of Minimally-Invasive Surgical Oncology, ASST Grande Ospedale Metropolitano Niguarda, Milan, Italy; 6 Department of Surgery, Pederzoli Hospital, Peschiera, Italy; 7 General Surgery, Department of Translational Research and New Technologies in Medicine and Surgery, University of Pisa, Pisa, Italy; 8 Department of Surgery, Poliambulanza Foundation Hospital, Brescia, Italy; 9 Department of Surgery, Ospedale Card. G. Panico, Tricase, Italy; 10 Hepato-Pancreato-Biliary Surgery and Liver Transplantation Unit, University of Modena and Reggio Emilia, Modena, Italy; 11 Division of HPB Minimally Invasive and Robotic Surgery, Department of Clinical Medicine and Surgery, Federico II University Hospital, Naples, Italy; 12 Hepatobiliary and Abdominal Transplantation Surgery, Department of Experimental and Clinical Medicine, Riuniti Hospital, Polytechnic University of Marche, Ancona, Italy; 13 Department of General Surgery, IRCCS, Azienda Ospedaliero-Universitaria di Bologna, Maggiore Hospital, Bologna, Italy; 14 Department of General and Oncological Surgery, “Umberto I” Mauriziano Hospital, Turin, Italy; 15 Department Clinical Medicine and Surgery, Federico II University of Naples, Via Pansini 5, 80131 Naples, Italy; 16 Digestive Surgery, Fondazione Policlinico Universitario A. Gemelli, IRCCS, Catholic University, Rome, Italy; 17 Division of Pancreatic Surgery, Department of Internal Medicine and Surgery (DIMEC), IRCCS, Azienda Ospedaliero Universitaria di Bologna Alma Mater Studiorum, University of Bologna, Bologna, Italy; 18 General and Oncology Surgery, Morgagni-Pierantoni Hospital, Forli, Italy; 19 Division of Oncologic Surgery and Robotics, Department of Oncology, Careggi University Hospital, Florence, Italy; 20 Department of Oncological Surgery Team 1, “Antonio Cardarelli” Hospital, Naples, Italy; 21 Hepatobiliary Surgery Unit Department of Medicine and Surgery, University of Parma, Parma, Italy; 22 Transplantation Department, S. Camillo-Forlanini Hospital, Rome, Italy; 23 Department of Hepato-Pancreatic-Biliary Surgery, General Regional Hospital “F. Miulli”, Acquaviva Delle Fonti, Bari, Italy; 24 4th Surgery Unit, Azienda ULSS2 Marca Trevigiana, Treviso, Italy; 25 Division of Hepatobiliary Surgical Oncology, Istituto Nazionale Tumori IRCCS Fondazione Pascale–IRCCS di Napoli, Naples, Italy; 26 Abdominal Surgery and Organ Transplantation Unit, ISMETT, Palermo, Italy; 27 Department of General Surgery and HPB Unit, Santa Chiara Hospital, Trento, Italy; 28 USL Toscana Sud Est, Misericordia Hospital, Grosseto, Italy; 29 General Surgery Unit, Infermi Hospital, Rimini, Italy; 30 Liver Transplant Center-General Surgery 2U, University of Turin, AOU Città della Salute e della Scienza di Torino, Turin, Italy; 31 Department of Surgery, Regional Hospital of Treviso, Treviso, Italy; 32 IRCCS Candiolo, Turin, Italy; 33 Ospedale Loreto Mare, Naples, Italy; 34 Department of Surgery, University Campus Bio-Medico of Rome, Fondazione Policlinico Universitario Campus Bio-Medico, Rome, Italy; 35 IRCCS Humanitas Research Hospital, Rozzano, Italy; 36 EndoCAS (Center for Computer Assisted Surgery), University of Pisa, Pisa, Italy; 37 Vita-Salute San Raffaele University, Milan, Italy

**Keywords:** Registry, Minimally invasive pancreatic resection, Minimally invasive distal pancreatectomy, Spleen-preserving minimally invasive distal pancreatectomy, Minimally invasive pancreatoduodenectomy, Minimally invasive total pancreatectomy, Minimally invasive central pancreatectomy

## Abstract

This retrospective analysis of the prospective IGOMIPS registry reports on 1191 minimally invasive pancreatic resections (MIPR) performed in Italy between 2019 and 2022, including 668 distal pancreatectomies (DP) (55.7%), 435 pancreatoduodenectomies (PD) (36.3%), 44 total pancreatectomies (3.7%), 36 tumor enucleations (3.0%), and 8 central pancreatectomies (0.7%). Spleen-preserving DP was performed in 109 patients (16.3%). Overall incidence of severe complications (Clavien–Dindo ≥ 3) was 17.6% with a 90-day mortality of 1.9%. This registry analysis provided some important information. First, robotic assistance was preferred for all MIPR but DP with splenectomy. Second, robotic assistance reduced conversion to open surgery and blood loss in comparison to laparoscopy. Robotic PD was also associated with lower incidence of severe postoperative complications and a trend toward lower mortality. Fourth, the annual cut-off of ≥ 20 MIPR and ≥ 20 MIPD improved selected outcome measures. Fifth, most MIPR were performed by a single surgeon. Sixth, only two-thirds of the centers performed spleen-preserving DP. Seventh, DP with splenectomy was associated with higher conversion rate when compared to spleen-preserving DP. Eighth, the use of pancreatojejunostomy was the prevalent reconstruction in PD. Ninth, final histology was similar for MIPR performed at high- and low-volume centers, but neoadjuvant chemotherapy was used more frequently at high-volume centers. Finally, this registry analysis raises important concerns about the reliability of R1 assessment underscoring the importance of standardized pathology of pancreatic specimens. In conclusion, MIPR can be safely implemented on a national scale. Further analyses are required to understand nuances of implementation of MIPR in Italy.

**Supplementary Information:**

The online version contains supplementary material available at 10.1007/s13304-023-01592-7.

## Introduction

Minimally invasive (MI) surgery was probably the greatest innovation in general surgery in the last century, and was immediately rewarding in operations requiring large incisions to perform low complexity surgeries.

Feasibility of MI pancreatic resections (MIPR) was shown over 25 years ago [1, 2]. However, MIPR had a slow implementation due to the intrinsic complexity of pancreatic surgery, often including multiple digestive reconstructions, and the lack of an obvious advantage in terms of improved outcomes [3]. Oncologic adequacy was an additional concern, especially for pancreatic cancer [4]. Thanks also to the advent of robotic technology, recent evidence shows that in selected patients, MIPR can offer clear advantages over the conventional open surgery [5–7]. These newer data come from centers of excellence that have surpassed a quite steep learning curve. In MI pancreatoduodenectomy (PD; MIPD), true proficiency is acquired after 250 procedures [8], making generalizability of results achieved by champion surgeons questionable.

The most effective tool to depict daily practice of MIPR is probably a prospective registry. Participation in national and international registries was also recommended by the International Evidence-Based Guidelines on Minimally Invasive Pancreatic Resections with the purpose of ensuring safe and wide expansion of MIPR [9]. At the moment, there are some generalist registries for pancreatic surgery [10], but only two registries for MI pancreatic surgery: the European Registry (http://www.e-mips.com/registry) and the Italian Registry (IGOMPIS: https://www.yoursuite.it/IGOMIPS/).

IGOMIPS is a prospective registry, established in 2019, that includes the majority of MIPR performed in Italy [11]. We herein report the first comprehensive analysis of the IGOMIPS registry with the aim of providing a snapshot of daily practice of MIPR in Italy. Detailed data are provided for distal pancreatectomy (DP) and PD, as these procedures account for over 90% of all MIPR.

## Materials and methods

### IGOMIPS

The Independent Ethics Committee of the Humanitas Institute (authorization number 2167) established the IGOMIPS registry in 2019 following approval. IGOMIPS was recorded in the Registry of Patient Registries (RoPR) of the Agency for Healthcare and Research and Quality, US Department of Health (Registry of Patient Registries. Content last reviewed April 2019 https://www.ahrq.gov/ropr/index.html).

A detailed description of IGOMIPS was previously reported [11]. Briefly, IGOMIPS is a prospective registry for MI pancreatic surgery capturing operative and outcome data up to 90-days after surgery. All Italian centers performing MI pancreatic surgery can apply to participate to IGOMIPS, following protocol approval by the local Ethical Committee. IGOMIPS has some unique features permitting several analyses that are not feasible in other registries. First, every procedure must be declared the day before surgery, so that concordance between planned and performed procedures can be defined. Second, IGOMIPS includes progressive case numbers for centers and individual surgeons, thus permitting to analyze performance based on experience. Third, participating centers are periodically audited to verify the quality of data and adherence to registry protocol.

### Study design

This study provides a retrospective review of MIPR performed at 34 IGOMPIS centers between September 2019 and June 2022. Comparisons and statistical analysis aim to provide insights on current practice of MIPR on a national basis.

Statistical computations were performed using the software STATA 17.0 (StataCorp. 2017. Stata Statistical Software: Release 15. College Station, TX: StataCorp LLC.). Descriptive and inferential statistics were carried out with the analytical models adequate for the type of variable studied (e.g., Mann–Whitney test, chi- square). Two-sided P values lower than 0.05 were considered statistically significant. All continuous variables were reported as the median and interquartile range (IQR).

### Data analysis

A per protocol analysis of MIPR enrolled in IGOMIPS up to June 2022 was performed. Other MI pancreatic surgeries (e.g., diagnostic laparoscopy, and biliary or gastric by-pass) were not included in the analysis.

Operations converted to open surgery were still analyzed as MI procedures (intention-to-treat analysis). We also reported rates of conversion to open surgery and agreement between planned and performed procedures.

### Center volume

The cut-off of ≥ 20 MIPR proposed by the International Evidence-Based Guidelines on Minimally Invasive Pancreatic Resections [9] was used to identify high-volume centers. Outcomes of centers above and below this cut-off were compared.

### Postoperative complications

Pancreas-specific complications were defined and graded as proposed by the International Study Group of Pancreatic Surgery [12–15]. Severity of all complications was assessed according to the Clavien and Dindo scale [16]. Severe complications were those with a score ≥ 3.

## Results

During the study period, a total of 1293 MI pancreatic procedures were reported to IGOMIPS from 34 Italian centers from 11 different regions accounting for 49,791,952 out of 59,030,133 inhabitants as of January 1st, 2022 (84.3%) (http://dati.istat.it/Index.aspx?DataSetCode=DCIS_POPRES, accessed January 15, 2023).

**Fig. 1:**
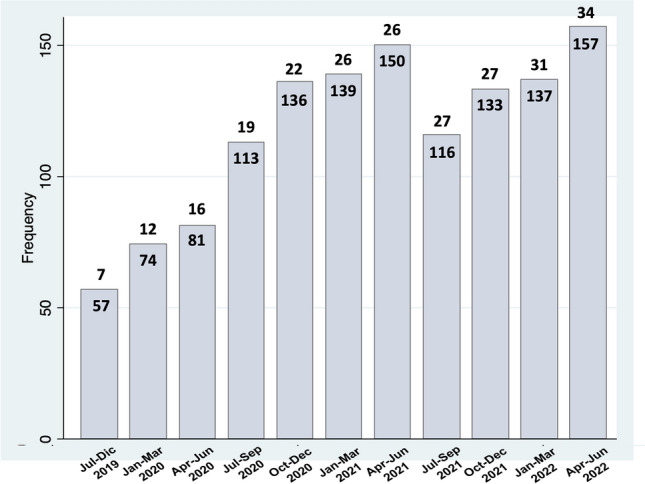
Number of cases reported (within columns) by trimester and number of active centers (above columns)

**Fig. 2:**
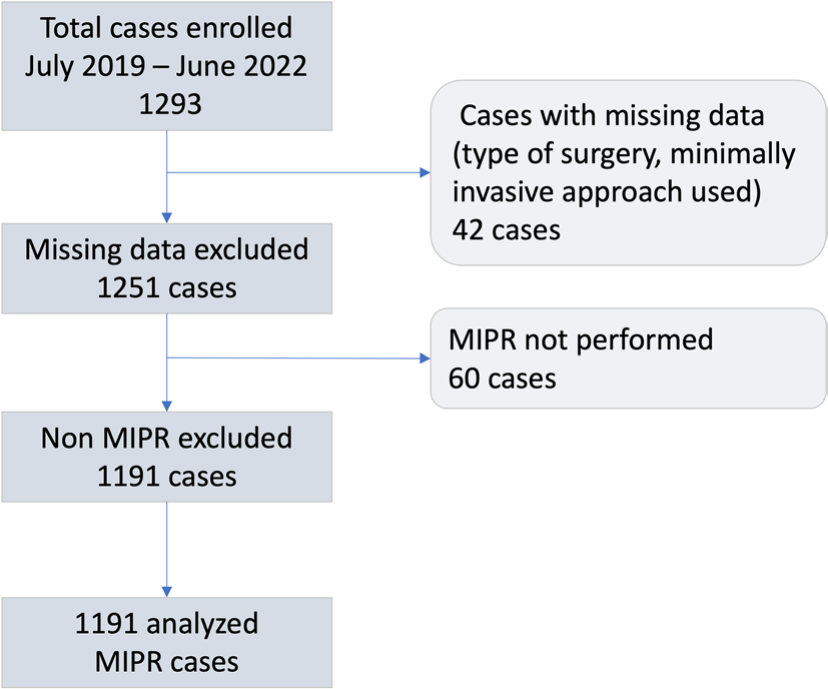
Study flowchart

Figure 1 shows case enrollment based on number of active centers and trimester. Figure 2 reports the study flowchart. Overall 1191 MIPR were analyzed following exclusion of 102 procedures, because an MIPR was not performed (*n* = 60; 4.0%) or due to missing data (*n* = 42; 4.2%). Number of procedures per center ranged from 1 to 161 with a median of 12 [49]. Most procedures (854/1191; 71.7%) were performed at nine centers performing ≥ 20 MIPR per year. Overall, the top five most active centers enrolled 618 MIPR (51.9%). DPWS was performed at all centers. PD was performed at 19 centers (55.9%) and SPDP at 21 centers (61.7%). All but one of the top five centers performed MIPD. Nineteen centers (55.9%) located in Northern Italy reported 743 MIPR (62.4%), 8 centers (23.5%) located in Central Italy reported 342 MIPR (28.7%), and 7 centers (20.6%) located in Southern Italy reported 106 MIPR (8.9%). Four of the five top recruiting centers were located in Northern Italy and reported a total of 461 MIPR. A single center, located in Central Italy, was the top recruiter with 155 MIPR (13.0%).

The MIPR most commonly reported to IGOMIPS was MIDP (*n* = 668; 55.7%), followed by MIPD (*n* = 435; 36.3%), total pancreatectomy (*n* = 44; 3.7%) tumor enucleation (*n* = 36; 3.0%), and central pancreatectomy (*n* = 8; 0.7%). MIDP was reported either with (DPWS) (*n* = 559; 46.7%) or without splenectomy (SPDP) (*n* = 109; 9.1%). Laparoscopy and robotic assistance were used in 635 (53.3%) and 510 (42.8%) MIPR, respectively. A hybrid laparoscopic (*n* = 42; 3.6%) or robotic (*n* = 4; 0.3%) was used in a minority of patients.

Overall, the use of laparoscopy was prevalent over robotic assistance in DPWS (69.9 vs 29.5%; *p* < 0.0001), but the opposite was true for PD (34.9% vs 55.6%; *p* < 0.0001). In SPDP (48.6% vs 50.5%), tumor enucleation (47.2% vs 52.8%), and TP (47.7% vs 50.0%), the use of laparoscopy and robotic assistance was similar. When the analysis was restricted to the 24 centers (70.6%) where a robotic platform was available and accessible for MIPR, the use of robotic assistance was prevalent for PD (36.6% vs 59.0%; *p* < 0.0001), SPDP (44.4% vs 55.6%; *p* = 0.230), TP (45.0% vs 55.0%; *p* = 0.527), and tumor enucleation (20.8% vs 79.2%; *p* = 0.004), while the prevalence of laparoscopy for DPWS was no longer evident (51.5% vs 48.5%). Central pancreatectomy was performed only in centers with robotic availability and all cases were performed under robotic assistance (Table 1).

**Table 1:** Laparoscopic, robotic, and hybrid Minimally Invasive Pancreatic Resections (MIPR)

		Laparoscopy	Robotic	Hybrid
*All MIPR*				
DPWS	559	391 (70.0%)	165 (29.5%)	3 (0.5%)
PD	435	152 (34.9%)	242 (55.6%)	38 (8.7%)
SPDP	109	53 (48.6%)	55 (50.5%)	1 (0.9%)
Total pancreatectomy	44	21 (47.7%)	22 (50.0%)	1 (2.3%)
Enucleation	36	17 (47.2%)	19 (52.8%)	0
Central Pancreatectomy	8	1 (12.5%)	7 (87.5%)	0
*MIPR reported by centers with a robotic platform*				
DPWS	340	175 (51.5%)	165 (48.5%)	0
PD	410	150 (36.6%)	242 (59.0%)	18 (4.4%)
SPDP	99	44 (44.4%)	55 (55.6%)	0
Total pancreatectomy	40	18 (45.0%)	22 (55.0%)	0
Enucleation	24	5 (20.8%)	19 (79.2%)	0
Central Pancreatectomy	7	0	7 (100%)	0

*DPWS* distal pancreatectomy with splenectomy, *PD* pancreaticoduodenectomy, *SPDP* spleen-preserving distal pancreatectomy

**Fig. 3:**
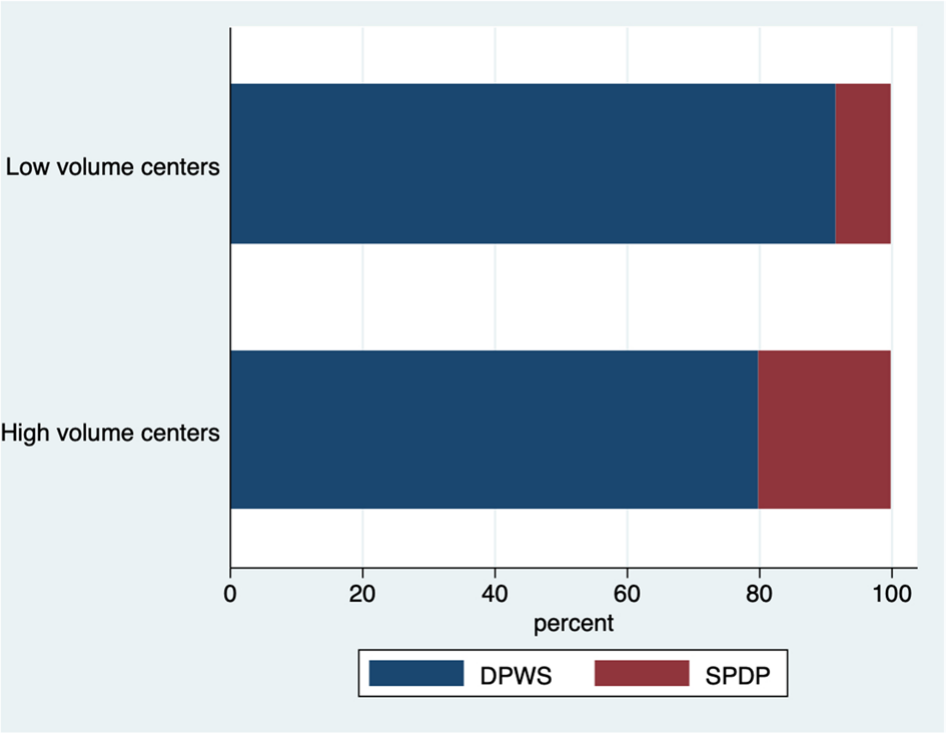
Proportion of DPWS and SPDP at high- and low-volume centers

Despite two high-volume centers did not perform this operation, the proportion of MIPD over all MIPR was higher at high-volume centers (303/516–58.7% vs 132/675–19.6%; *p* < 0.0001). Also, the proportion of SPDP was higher at high-volume centers (91/455–20.0% vs 18/213–8.4%; *p* < 0.001) (Fig. 3). PD and SPDP were mostly performed by the same surgeon, but the fraction of procedures performed by the same surgeon was higher at low-volume centers (MIPD: 93.7% vs 83.6%; *p* = 0.017) (MIDP: 96.1% vs 68.8%; *p* = 0.038) (Table 2).

**Table 2:** Proportion of Minimally Invasive Pancreatic Resections (MIPR) performed by a single surgeon at each center

	Procedures	Performed by a single surgeon mean proportion (range)
*All MIPR*		
DPWS	553	78.9% (25.0–100)
SPDP	107	84.5% (42.3–100)
PD	430	90.4% (51.3–100)
Total pancreatectomy	43	96.7% (66.7–100)
Enucleation	36	91.9% (40.0–100)
Central pancreatectomy	8	100%
*Laparoscopic MIPR*		
DPWS	386	78.2% (25.0–100)
SPDP	53	86.8% (50–100)
PD	150	91.4% (37.5–100)
*Robotic MIPR*		
DPWS	164	79.1% (34.38–100)
SPDP	5	90.4 (42.3–100)
PD	239	91.7% (51.3–100)
*MIPR at low-volume centers*		
DPWS	195	82.2% (35.3–100)
SPDP	17	95.8% (50–100)
PD	88	93.1% (62.5–100)
*MIPR at high-volume centers*		
DPWS	358	69.8% (25.0–100)
SPDP	90	72.2% (42.3–100)
PD	342	86.0% (51.3–100)

Data on operating surgeon available for 1177 MIPR

*DPWS* distal pancreatectomy with splenectomy, *SPDP* spleen-preserving distal pancreatectomy, *PD* pancreaticoduodenectomy

### Distal pancreatectomy

DPWS was performed at all the 34 IGOMIPS centers, while SPDP was performed at 21 centers (61.7%). Severe postoperative complications occurred in 69 patients (10.3%) and 2 patients died (0.3%). Repeat surgery and hospital readmission were required in 15 (2.4%) and 64 (9.5%) patients, respectively. Median length of hospital stay was 7 days [6–10].

Table 3 provides a summary of baseline characteristics, intraoperative data, and postoperative outcome of patients undergoing either DPWS or SPDP.

**Table 3:** Baseline characteristics, intraoperative outcome measures, and early postoperative results of Distal Pancreatectomy with Splenectomy (DPWS) and Spleen-Preserving Distal Pancreatectomy (SPDP)

	DPWS (*n* = 559)	SPDP (*n* = 109)
Median age [IQR], years	63.5 (54–73)	56 (42–70)
Median BMI [IQR], Kg/m^2^	24.8 (22.5–27.9)	25 (22–29)
Female, *n* (%)	303 (54.2)	60 (54.5)
ASA ≥ 3, *n* (%)	179 (32.3)	34 (31.2)
Previous abdominal surgery, *n* (%)	251 (45.0)	49 (44.5)
*Minimally invasive technique*^§^		
Robotic, *n* (%)	165 (29.5)	55 (50.5)
Laparoscopic, *n* (%)	391 (70.0)	53 (48.6)
Other, *n* (%)	3 (0.5)	1 (0.9)
Median operative time [IQR], min	252 (210–325)	240 (195–317)
Median estimated blood loss [IQR], mL	100 (80–250)	100 (50–189)
Preservation of splenic vessels	–	78 (84.7)
*Pancreatic stump closure* *n* (%)^ç^		
Stapled	354 (80.6)	73 (76.8)
Reinforced stapling	155 (45)	38 (53.1)
Suture	43 (9.8)	9 (9.5)
Harmonic shears	42 (9.6)	13 (13.7)
Conversion to open surgery, *n* (%)	43 (7.7)	1 (0.9)
*Clinically relevant postoperative pancreatic fistula*, *n* (%) [12]	98 (18.4)	16 (15.5)
Grade B	105	16
Grade C	1	0
Delayed gastric emptying, *n* (%)	9 (1.7)	1 (0.9)
Postpancreatectomy hemorrhage *n* (%)	14 (2.6)	5 (4.8)
Wound infection, *n* (%)	8 (1.5)	0
Abdominal fluid collection, *n* (%)	96 (18.0)	17 (16.5)
Sepsis, *n* (%)	28 (5.2)	1 (0.9)
Severe postoperative complications, *n* (%)	60 (10.4)	9 (8.2)
Reintervention, *n* (%)	11 (2.1)	4 (4.0)
Readmission, *n* (%)	57 (11.1)	9 (9.0)
Median length of hospital stay [IQR], days	7 (6–10)	7 (5–11)
Postoperative mortality, *n* (%)	2 (0.3)	0
Malignant histology, *n* (%)	232 (43.3)	–
RAMPS, *n* (%)*	101 (45.9)	–
Associated organ resections, *n* (%)*	18 (7.8)	–
Associated vascular resection, *n* (%)*	14 (6.0)	–
*N* of resected lymph nodes*	23 (15–34)	–
R1 resection rate*	41 (21.7)	–

^ç^Data available for 95 SPDP and 439 DPWS; °data available for 92 patients. ^§^Data available for 108 SPDP, and 556 DPWS. *Data calculated only for malignant pancreatic lesions

*BMI* body mass index, *ASA* American Society of Anesthesiologists, *RAMPS* radical anterograde modular pancreatosplenectomy

Radical Anterograde Modular Pancreatosplenectomy (RAMPS) was performed in 101 of 232 DPWS for pancreatic cancer (45.9%). Ninety-five patients had an anterior RAMPS (94.1%) and 6 had a posterior RAMPS (5.9%). Fourteen patients had a vascular resection (9.3%). Excluding posterior RAMPS, 18 patients required resection of an extrapancreatic organ (7.8%). Patients operated at high-volume center were more likely to have received neoadjuvant chemotherapy (21.6% versus 8.9%, *p* = 0.015) and showed a trend for having undergone more frequently RAMPS (51.0% versus 36.0%, *p* = 0.034).

Data on modality of pancreatic transection were available for 534 patients (80.0%). A stapler was used to divide and seal the pancreatic stump in most MIDP (*n* = 427, 80.0%). In the remaining patients, the pancreas was either divided by harmonic shears alone (*n* = 55; 10.3%) or by an energy device plus fish-mouth sutures (*n* = 52; 9.7%).

Overall, 114 of 668 patients developed a clinically relevant postoperative pancreatic fistula (17.0%) (grade B: 113; 16.9%) (grade C: 1; 0.1%). Closure of pancreatic remnant by harmonic shears alone was associated with higher incidence of clinically relevant postoperative pancreatic fistula (harmonic shears: 36.4%; stapler: 18.4%; energy device plus sutures: 14.0%; *p* = 0.004), as well as of severe postoperative complications (harmonic shears: 27.3%; energy device plus sutures: 13.5%; stapler: 8.7% *p* < 0.0001). Neither staple line reinforcement (*n* = 188; 46.7%) nor the application of sealants on the pancreatic stump (*n* = 134; 20.7%) affected incidence of clinically relevant postoperative pancreatic fistula as well as of severe complications.

Out of 548 planned DPWS, 526 (96.0%) were actually performed. The 22 (3.9%) unplanned procedures were 6 SPDP, 9 tumor enucleations, 1 PD, and 6 total pancreatectomies. Conversion to open surgery was required in 45 of these patients (8.2%). On the other hand, 33 patients had an unplanned DPWS instead of SPDP (*n* = 25), tumor enucleation (*n* = 4), PD (*n* = 1), total pancreatectomy (*n* = 2), and central pancreatectomy (*n* = 1).

Out of 127 planned SPDP, 96 (75.6%) were actually performed. The 31 (24.4%) unplanned procedures were 25 DPWS, 4 tumor enucleations, and 2 central pancreatectomies. Conversion to open surgery was required in two of these patients (1.6%). On the other hand, 13 patients had an unplanned SPDP instead of DPWS (*n* = 6), enucleation (*n* = 4), and central pancreatectomy (*n* = 3).

Most MIDP were performed laparoscopically (*n* = 444; 66.4%), but robotic assistance was prevalent for SPDP (50.5% in SPDP versus 29.5% in DPWS; *p* < 0.001). Splenic vessels were preserved along with the spleen in 78 patients (84.7%).

Conversion to open surgery was required in 48 MIDP (6.6%), and occurred more frequently in DPWS (*n* = 43; 7.7%) than in SPDS (*n* = 1; 0.9%) (*p* = 0.009). However, 25 patients planned for SPDP underwent DPWS. In an intention-to-treat analysis, conversion rate for planned DPWS was 8.2% (*n* = 45) and for planned for SPDP 1.6% (*n* = 2) (*p* = 0.008).

**Fig. 4:**
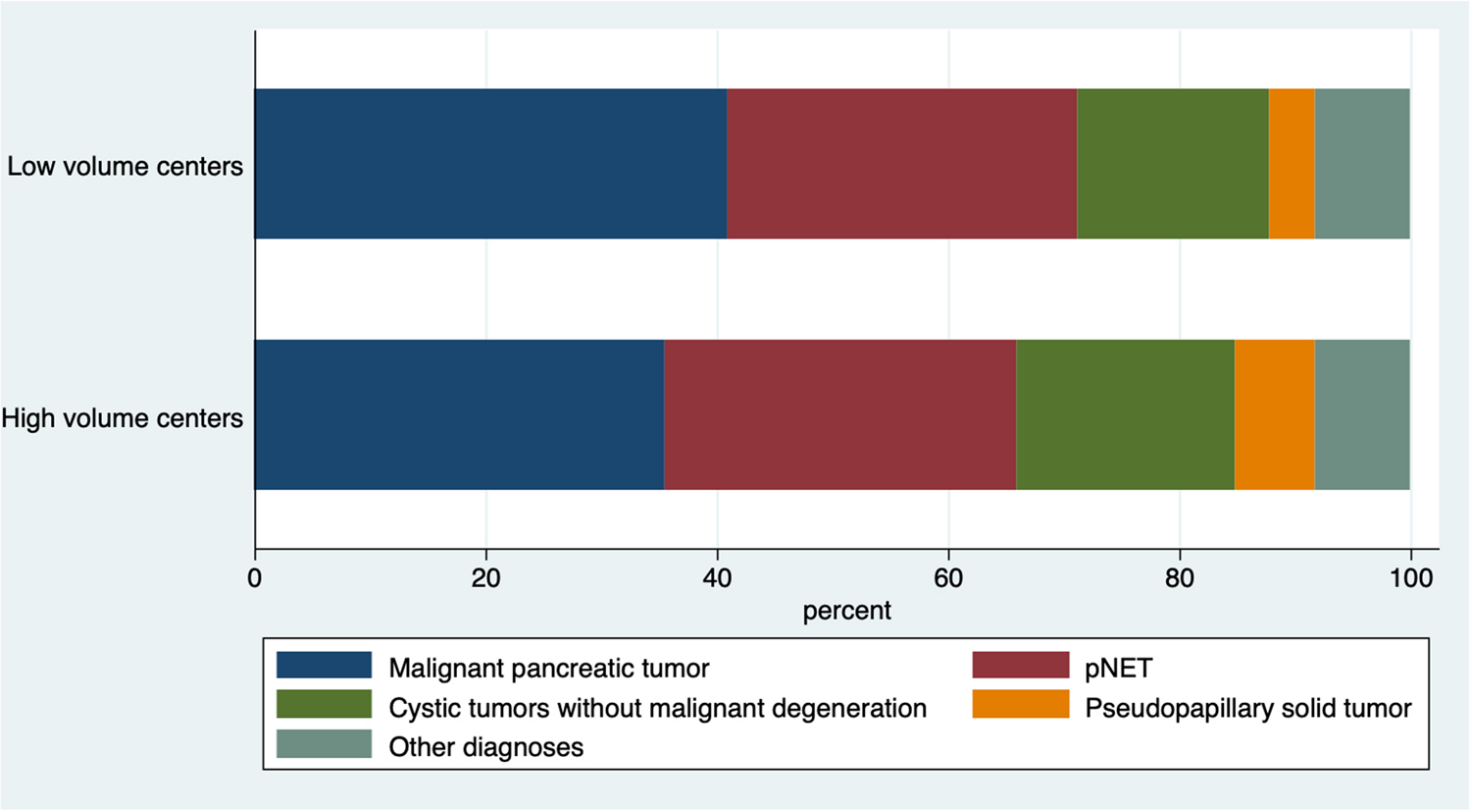
Distribution of tumor types at high- and low-volume centers. The seemingly higher proportion of malignant histology at low-volume centers (however, not reaching statistical significance) is explained by the higher proportion of SPDP at high-volume centers. *pNET* pancreatic neuroendocrine tumor

Histology was available for 639 MIDP (95.6%). Pancreatic cancer was the leading diagnosis (*n* = 237; 37.0%), followed by neuroendocrine tumor (*n* = 198; 31.2%), mucinous cystoadenoma (*n* = 52; 8.2), intraductal papillary mucinous neoplasm (IPMN) without malignant degeneration (*n* = 44; 6.9%), solid pseudopapillary tumor (*n* = 38; 5.9%), serous cystadenoma (*n* = 20; 3.1%), and metastasis from renal carcinoma (*n* = 11; 1.7%). The remaining 39 patients had less frequent tumor types (6.1%). Frequency of individual tumor types was similar between high- and low-volume centers (Fig. 4).

### Laparoscopic versus robotic DPWS

As shown in Table 4, compared to laparoscopy, robotic DPWS was associated with longer median operative time (280 [120] versus 240 [110] minutes; *p* < 0.0001) and longer median duration of hospital stay (8 [5] versus 7 [4] days; *p* = 0.002), but lower rates of conversion to open surgery (10.1% vs 2.5%; *p* = 0.002) and lower estimated blood loss (100 [150] versus 150 [200] mL; *p* < 0.0001). Laparoscopic DPWS also showed a trend to statistical significance for lower incidence of intraabdominal fluid collections (16.1% vs 23.2%; *p* = 0.053).

**Table 4:** Baseline characteristics, intraoperative outcome measures, and early postoperative results of laparoscopic versus robotic Distal Pancreatectomy with Splenectomy (DPWS)

	Laparoscopic DPWS *n* = 391	Robotic DPWS *n* = 165	p
Median age [IQR], years	64 (55–74)	62 (54–72)	0.207
BMI, kg/m^2^	25.0 (22.4–27.8)	24.3 (23.0–27.8)	0.972
Female, *n*	212 (54.2%)	89 (53.9%)	0.952
ASA ≥ 3, *n* (%)	127 (32.8%)	50 (30.3%)	0.562
Previous abdominal surgery, *n* (%)	179 (45.9%)	70 (42.4%)	0.452
Median operative time [IQR], minutes	240 (196–306)	280 (240–360)	0.0001
Median estimated blood loss [IQR], mL	150 (100–300)	100 (50–200)	0.0001
Conversion to open surgery, *n* (%)	39 (10.1%)	4 (2.5%)	0.002
Clinically relevant postoperative pancreatic fistula, *n* (%)	70 (18.7%)	27 (17.4%)	0.726
Delayed gastric emptying, *n* (%)	7 (1.9%)	2 (1.3%)	0.638
Postpancreatectomy hemorrhage *n* (%)	10 (2.7%)	4 (2.6%)	0.952
Wound infection, *n* (%)	7 (1.9%)	1 (0.7%)	0.293
Abdominal collection, *n* (%)	60 (16.1%)	36 (23.2%)	0.053
Sepsis, *n* (%)	20 (5.3%)	8 (5.2%)	0.931
Severe postoperative complications	43 (11.0%)	17 (10.3%)	0.809
Reintervention, *n* (%)	6 (1.7%)	5 (3.2%)	0.260
Readmission, *n* (%)	37 (10.4%)	20 (13.2%)	0.347
Median length of hospital stay [IQR], days	7 (6–10)	8 (6–11)	0.002
Postoperative mortality, *n* (%)	1 (0.3%)	1 (0.7%)	0.529
Malignant histology, *n* (%)	162 (43.4%)	68 (42.5%)	0.842
RAMPS, *n* (%)*	72 (46.7%)	28 (43.7%)	0.685
Associated organ resections, *n* (%)*	14 (8.6%)	4 (5.9%)	0.477
Associated vascular resection, *n* (%)*	13 (8.0%)	1 (1.5%)	0.058
N of resected LN *	23 (16–38)	21.5 (14–32.5)	0.394
R1 resection rate *	37 (28.0%)	4 (7.3%)	0.002

*Data calculated only for malignant pancreatic lesions

*BMI* body mass index, *ASA* American Society of Anesthesiologists, *RAMPS* radical anterograde modular pancreatosplenectomy

In patients with pancreatic cancer, despite similar T stage, nodal status, and number of examined lymph nodes, robotic DPWS was associated with lower rates of R1 resection (7.3% vs 28.0%; *p* = 0.002).

### Laparoscopic versus robotic SPDP

As shown in Table 5, laparoscopic and robotic SPDP were similar in all respects, including need for unplanned splenectomy (laparoscopic: 22.7% vs robotic: 18.5%; *p* = 0.572).

**Table 5:** Baseline characteristics, intraoperative outcome measures, and early postoperative results of laparoscopic versus robotic Spleen-Preserving Distal Pancreatectomy  (SPDP)

	Laparoscopic SPDP *n* = 53	Robotic SPDP *n* = 55	p
Median age [IQR], years	56 (38–70)	54 (42–69)	0.953
BMI, Kg/m^2^	25 (22–28)	25 (22–29)	0.433
Female, *n*	32 (60.4%)	28 (50.9%)	0.322
ASA ≥ 3, *n* (%)	19 (35.8%)	14 (25.9%)	0.266
Previous abdominal surgery, *n* (%)	24 (45.3%)	25 (45.4%)	0.986
Median operative time [IQR], minutes	226.5 (170–315)	255.0 (220–331)	0.142
Median estimated blood loss [IQR], mL	100 (50–200)	100 (50–150)	0.227
Preservation of splenic vessels°	38 (86.4%)	39 (83.0%)	0.655
Conversion to open surgery, *n* (%)	1 (1.9%)	0	0.315
Clinically relevant postoperative pancreatic fistula, *n* (%)	10 (19.6%)	6 (11.8%)	0.276
Delayed gastric emptying, *n* (%)	1 (1.9%)	0	0.315
Postpancreatectomy hemorrhage, *n* (%)	3 (5.9%)	2 (3.9%)	0.647
Wound infection, *n* (%)	0	0	
Abdominal collection, *n* (%)	12 (23.5%)	5 (9.8%)	0.063
Sepsis, *n* (%)	1 (1.9%)	0	0.315
Severe postoperative complications (Clavien–Dindo ≥ 3), *n* (%) [14]	6 (11.3%)	3 (5.4%)	0.270
Reintervention, *n* (%)	2 (3.8%)	2 (3.6%)	1
Readmission, *n* (%)	5 (9.4%)	4 (7.3%)	0.727
Median length of hospital stay [IQR], days	7 (6–12)	6.5 (5–9)	0.061
Postoperative mortality, *n* (%)	0	0	

°Data available for 92 cases

*BMI* body mass index, *ASA* American Society of Anesthesiologists

### Pancreatoduodenectomy

Table 6 provides a summary of baseline characteristics, intraoperative data, and postoperative outcome for patients undergoing MIPD.

**Table 6:** Baseline characteristics, intraoperative outcome measures, and early postoperative results of Minimally Invasive Pancreatoduodenectomy (MIPD)

	Total *N* = 335
Median age [IQR], years	68 (60–75)
Median BMI [IQR], Kg/m^2^	24.4 (22.4–27.0)
Female, *n* (%)	204 (46.9)
ASA ≥ 3, *n* (%)	181 (41.7)
Previous abdominal surgery, *n* (%)	158 (36.5)
Median operative time [IQR], minutes	490 (425–570)
Median estimated blood loss [IQR], mL	200 (124–400)
Hard stump consistence, *n* (%)	140 (34.4)
*Pancreatic anastomosis*	
Pancreatojejunostomy	387 (93.8)
Pancreatojejunostomy on a Roux-en-Y jejunal loop	13 (3.1)
Pancreatogastrostomy	13 (3.1)
Duct stent, *n* (%)	226 (55.1)
Pylorus preserving, *n* (%)	227 (55.2)
Conversion to open surgery, *n* (%)	45 (10.3)
*Clinically relevant postoperative pancreatic fistula*, *n* (%) [12]	73 (18.7)
Grade B	61
Grade C	12
Biliary leak, *n* (%)	27 (6.9)
Gastro-enteric leak, *n* (%)	11 (2.8)
Chyle leak, *n* (%)	17 (4.4)
Delayed gastric emptying, *n* (%)	74 (19.0)
Postpancreatectomy hemorrhage *n* (%)	54 (13.8)
Wound infection, *n* (%)	18 (4.6)
Abdominal fluid collection, *n* (%)	110 (28.2)
Sepsis, *n* (%)	49 (12.6)
Severe postoperative complications, *n* (%)	121 (27.8)
Reintervention, *n* (%)	48 (12.6)
Readmission, *n* (%)	49 (13.0)
Median length of hospital stay [IQR], days	13 (8–22)
Postoperative mortality, *n* (%)	21 (4.8)
Malignant histology, *n* (%)	326 (81.7)
Associated organ resections, *n* (%)*	5 (1.2)
Associated vein resection, *n* (%)*	20 (4.7)
Number of examined lymph nodes, *n* (%)*	26 (18–36)
R1 resection rate, *n* (%)*	60 (20.8)

*Data calculated only for malignant pancreatic/peri-pancreatic lesions

*BMI* body mass index, *ASA* American Society of Anesthesiologists

The pylorus was preserved in 227 MIPD (55.2%), vein resection and reconstruction were performed in 20 procedures (4.6%), and resection of adjacent organs in 5 operations (1.2%). Details of digestive reconstruction were available for 413 patients. A single jejunal loop was employed in 379 MIPD (91.7%) and the pancreatic anastomosis was a pancreatico-jejunostomy in 400 patients (96.8%) and a pancreatogastrostomy in 13 (3.1%). Pancreatico-jejunostomy was performed end-to-side in 379 of 400 MIPD (94.7%), mostly using either a Blumgart (*n* = 60; 15.0%) or a modified Blumgart technique (*n* = 108; 27.0%). A double layer of sutures was used in 342 pancreatico-jejunostomy (85.5%) and a duct stent was used in 226 patients (55.1%).

Conversion to open surgery was required in 45 MIPD (10.3%). Median length of hospital stay was 13 days [8–22]. Severe postoperative complications occurred in 121 patients (27.8%) and 21 patients died (4.8%). Clinically relevant postoperative pancreatic fistula was diagnosed in 73 patients (17.1%) (grade B: 61; 14.3%) (grade C: 12; 2.8%). Repeat surgery and hospital readmission were required in 48 (12.6%) and 49 (13.0%) patients, respectively. Two patients (0.4%) required more than one reintervention.

Out of 437 planned MIPD, 422 (96.6%) were performed. The 15 (3.4%) unplanned procedures were one DPWS, one tumor enucleation, and 13 total pancreatectomies. Conversion to open surgery was required in 44 of these patients (10.1%). On the other hand, 13 patients had an unplanned MIPD instead of DPWS (*n* = 4), tumor enucleation (*n* = 3), total pancreatectomy (*n* = 4), central pancreatectomy (*n* = 1), and ampullectomy (*n* = 1).

Most PD were performed at high-volume centers (*n* = 303; 69.3%) using robotic assistance (*n* = 242; 55.6%). Laparoscopy was used in 152 PD (34.9%). When the analysis was restricted to the 16 centers that have a robot, implementation of PD increased to 44.6% and the use of robotic assistance to 59.0%. Adoption of robotic assistance increased over time. It was 7.1% in 2019, 47.6% in 2020, 57.7% in 2021, and 71.1% in 2022 (*p* < 0.0001). A hybrid technique, mostly using a mini-incision for some open anastomosis, was used in 37 additional laparoscopic (8.5%) and 4 robotic PD (0.9%), respectively.

Histology was available for 399 PD (91.7%). Pancreatic cancer was the leading diagnosis (*n* = 193; 48.4%), followed by adenocarcinoma of the ampulla of Vater (*n* = 77; 19.4%), distal common bile duct cancer (*n* = 42; 10.4%), and duodenal cancer (*n* = 14; 3.5%). There were also 25 neuroendocrine tumors (6.4%) and 17 benign cystic tumors (3.8%). The remaining 31 patients had less frequent tumor types (8.1%). Frequency of individual tumor types was similar between high- and low-volume centers.

### Laparoscopic versus robotic PD

As shown in Table 7, comparing laparoscopic PD and robotic PD showed that the latter was performed in younger patients (67 years versus 69 years; *p* = 0.017) and in persons with a lower prevalence of previous abdominal surgery (31.9% versus 42.1%; *p* = 0.041). However, the prevalence of patients with ASA score ≥ 3 (47.1% versus 36.8%; *p* = 0.045) was higher in robotic PD. Robotic PD was associated with lower estimated blood loss (200 [200] ml versus 300 [350] ml; *p* = 0.0001), fewer conversions to open surgery (4.8% versus 17.8%; *p* < 0.0001), and reduced rate of severe postoperative complications (23.1% versus 33.0%; *p* = 0.024). Postoperative mortality was also reduced after robotic PD (3.3% versus 7.2%; *p* = 0.076), despite the difference did not reach statistical significance. Laparoscopic PD was associated with fewer intraabdominal fluid collections (21.4% versus 34.6%; *p* = 0.008).

**Table 7:** Baseline characteristics, intraoperative outcome measures, and early postoperative results of laparoscopic versus robotic pancreaticoduodenectomy (PD)

	Laparoscopic pancreaticoduodenectomy *N* = 152	Robotic pancreaticoduodenectomy *N* = 242	*p*
Median age [IQR], years	69 (61–77)	67 (59–74)	0.017
BMI, Kg/m^2^	24.5 (22.4–27.3)	24.2 (22.3–26.5)	0.290
Female, *n*	65 (42.8%)	124 (51.2%)	0.101
ASA ≥ 3, *n* (%)	56 (36.8%)	114 (47.1%)	0.045
Previous abdominal surgery, *n* (%)	64 (42.1%)	77 (31.9%)	0.041
Median operative time [IQR], minutes	500 (420–560)	487 (430–575)	0.244
Median estimated blood loss [IQR], mL	300 (150–500)	200 (100–300)	0.0001
Hard stump consistence, *n* (%)	63 (44.1%)	67 (29.8%)	0.005
Pylorus preservation, *n* (%)	125 (85.6%)	45 (19.8%)	< 0.0001
Conversion to open surgery, *n* (%)	26 (17.8%)	11 (4.8%)	< 0.0001
Clinically relevant postoperative pancreatic fistula, *n* (%)	19 (13.6%)	45 (21.0%)	0.075
Biliary leak, *n* (%)	10 (7.1%)	13 (6.1%)	0.690
Gastro-enteric leak, *n* (%)	4 (2.9%)	6 (2.8%)	0.976
Chyle leak, *n* (%)	5 (3.6%)	11 (5.1%)	0.487
Delayed gastric emptying, *n* (%)	27 (19.3%)	46 (21.5%)	0.615
Postpancreatectomy hemorrhage *n* (%)	22 (15.6%)	30 (14.0%)	0.680
Wound infection, *n* (%)	7 (5.0%)	7 (3.3%)	0.414
Abdominal fluid collection, *n* (%)	30 (21.4%)	74 (34.6%)	0.008
Sepsis, *n* (%)	14 (10.0%)	33 (15.4%)	0.142
Severe postoperative complications, *n* (%)	51 (33.5%)	56 (23.1%)	0.024
Reintervention, *n* (%)	18 (13.0%)	25 (12.0%)	0.765
Readmission, *n* (%)	19 (13.8%)	23 (11.3%)	0.501
Median length of hospital stay [IQR], days	12 (8–21)	14 (9–23)	0.099
Postoperative mortality, *n* (%)	11 (7.2%)	8 (3.3%)	0.076
Malignant histology, *n* (%)	128 (87.7%)	169 (78.2%)	0.586
Associated organ resections, *n* (%)*	3 (2.0%)	1 (0.4%)	0.139
Associated vascular resection, *n* (%)*	4 (2.7%)	16 (6.8%)	0.074
*N* of examined LN, *n* (%) *	22 (16–30)	30 (21–41)	0.0001
R1 resection rate, *n* (%) *	16 (14.0%)	36 (23.7%)	0.050

*Data calculated only for malignant pancreatic/peri-pancreatic lesions

*BMI* body mass index, *ASA* American Society of Anesthesiologists

In patients operated for a malignant tumor, with an identical prevalence of T3/4 tumors and a higher prevalence of node positive patients in robotic PD (68.7% versus 57.3%; *p* = 0.049), robotic PD showed a higher median number of examined lymph nodes (30 [20] versus 21.5 [14]; *p* = 0.0001) but a higher rate of R1 resection (23.7% versus 14.0%; *p* = 0.050).

### Results of MIPR based on center volume

Results of MIPR based on center volume are presented in Table 8. Center volume had an effect on selected outcome measures.

**Table 8:** Baseline patient characteristics and outcomes of Minimally Invasive Pancreatic Resections (MIPR) based on the cut-off of ≥ 20 procedures per year

	DP			DPWS			SPDP			PD		
	≥ 20 MIPR	< 20 MIPR	*p*	≥ 20 MIPR	< 20 MIPR	*p*	≥ 20 MIPR	< 20 MIPR	*p*	≥ 20 MIPD	< 20 MIPD	*p*
	*n *= 455	*n * = 213		*n* = 364	*n* = 195		*n* = 91	*n* = 18		*n* = 303	*n* = 132	
Median age [IQR], years	62 (51–71)	65 (55–75)	**0.01**	62.5 (53–72)	65 (57–75)	**0.0195**	56 (40–68)	64 (48–77)	0.075	69 (61–76)	64.5 (58–73)	**0.016**
Median BMI [IQR], kg/m^2^	24.6 (22.2–27.5)	25.7 (22.9–28.9)	**0.026**	24.5 (22.4–27.4)	25.7 (22.9–28.8)	**0.015**	25.2 (22.2–28.1)	25.3 (20.8–31.1)	0.971	24.5 (22.6–27.2)	23.9 (22.0–26.0)	**0.024**
Female, *n* (%)	248 (54.5)	115 (54.0)	0.901	200 (54.9)	103 (52.8)	0.631	48 (52.7)	12 (66.7)	0.278	138 (45.5)	66 (50.0)	0.392
ASA ≥ 3, *n* (%)	130 (28.8)	83 (39.1)	**0.008**	105 (29.1)	74 (38.1)	**0.029**	25 (27.8)	9 (50.0)	0.064	139 (45.9)	42 (32.1)	**0.007**
Previous abdominal surgery, *n* (%)	213 (46.8)	87 (41.0)	0.163	171 (47.0)	80 (41.2)	0.194	42 (46.1)	7 (38.9)	0.571	118 (38.9)	40 (30.8)	0.105
Median operative time [IQR], min	256 (210–333)	240 (195–300)	**0.006**	258.5 (211–335)	240 (200–300)	**0.007**	242.5 (200–330)	230 (182–280)	0.188	493 (440–565)	485 (410–575)	0.400
Median estimated blood loss [IQR], mL	100 (100–220)	100 (50–200)	0.233	100 (100–250)	100 (50–225)	0.111	100 (50–194)	100 (50–150)	0.949	200 (100–300)	300 (150–500)	**0.0001**
Conversion to open surgery, *n* (%)	27 (5.9)	17 (8.0)	0.320	27 (7.4)	16 (8.2)	0.739	0	1 (5.6)	**0.024**	15 (4.9)	30 (22.7)	**0.0001**
*Clinically relevant postoperative pancreatic fistula*, *n* (%) [12]	84 (19.0)	30 (15.6)	0.314	68 (19.1)	30 (16.9)	0.536	16 (18.2)	0	0.072	53 (18.9)	20 (18.2)	0.865
Grade B	84	29		68 (100)	29 (96.7)		16 (100)	0		46 (86.8)	15 (75.0)	
Grade C	0	1		0	1 (3.3)		0	0		7 (13.2)	5 (25.0)	
Biliary leak, *n* (%)	–	–	–	–	–	–	–	–	–	16 (5.7)	11 (10.0)	0.134
Gastro-enteric leak, *n* (%)	–	–	–	–	–	–	–	–	–	10 (3.6)	1 (0.9)	0.153
Chyle leak, *n* (%)	8 (1.8)	2 (1.0)	0.477	8 (2.2)	2 (1.1)	0.369	0	0	–	9 (3.2)	8 (7.3)	0.077
Delayed gastric emptying, *n* (%)	7 (1.6)	3 (1.6)	0.987	6 (1.7)	3 (1.7)	0.997	1 (1.1)	0	0.687	61 (21.8)	13 (11.8)	**0.024**
Postpancreatectomy hemorrhage, *n* (%)	17 (3.8)	2 (1.0)	0.058	12 (3.4)	2 (1.1)	0.127	5 (5.7)	0	0.344	40 (14.3)	14 (12.6)	0665
Wound infection, *n* (%)	5 (1.1)	3 (1.6)	0.653	5 (1.4)	3 (1.7)	0.798	0	0	–	11 (3.9)	7 (6.4)	0.302
Abdominal fluid collection, *n* (%)	75 (17.0)	38 (19.8)	0.393	58 (16.4)	38 (21.5)	0.151	17 (19.3)	0	0.062	83 (29.6)	27 (24.5)	0.314
Sepsis, *n* (%)	22 (5.0)	7 (3.6)	0.464	21 (5.9)	7 (3.9)	0.340	1 (1.1)	0	0.678	39 (13.9)	10 (9.1)	0.195
Severe postoperative complications, *n* (%)	53 (11.6)	16 (7.5)	0.102	44 (12.1)	16 (8.2)	0.158	9 (9.9)	0	0.164	89 (29.4)	32 (24.2)	0.272
Reintervention, *n* (%)	12 (2.8)	3 (1.6)	0.397	9 (2.6)	2 (1.2)	0.287	3 (3.4)	1 (7.8)	0.466	34 (12.4)	14 (13.0)	0.883
Readmission, *n* (%)	50 (11.8)	16 (8.6)	0.247	41 (12.1)	16 (9.2)	0.327	9 (10.3)	0	0.224	37 (13.8)	12 (13.8)	0.482
Median length of hospital stay [IQR], days	7 (6–10)	7 (6–9)	0.954	7 (6–10)	7 (6–10)	0.111	7 (5–11)	7 (5–9)	0.421	14 (8–23)	11 (8–16)	**0.003**
Postoperative mortality, *n* (%)	0	2 (0.9)	**0.038**	0	2 (1.0)	0.053	0	0	–	14 (4.6)	7 (5.3)	0.760
Malignant histology, *n* (%)	157 (35.7)	81 (40.7)	0.224	153 (43.3)	79 (43.2)	0.969	–	–	–	237 (82.6)	89 (79.5)	0.470
Associated organ resections, *n *(%)*	13 (8.3)	5 (6.2)	0.560	13 (8.5)	5 (6.3)	0.559	–	–	–	2 (0.9)	2 (2.2)	0.307
Associated vascular resection, *n* (%)*	12 (7.6)	2 (2.5)	0.108	12 (7.8)	2 (2.5)	0.107	–	–	–	17 (7.2)	3 (3.4)	0.200
N of examined lymph nodes, *n* (%)*	26 (18–38)	15 (11–23)	**0.0001**	26 (18–38)	15 (11–23)	**0.0001**	–	–	–	26 (18–35)	30 (20–40)	0.161
R1 resection rate, *n* (%)*	36 (26.7)	5 (8.3)	**0.004**	36 (27.5)	5 (8.6)	**0.004**	–	–	–	52 (23.2)	8 (12.5)	0.063

Bold values indicate statistical significance of *p* values

*DP* distal pancreatectomy, *DPWS* distal pancreatectomy with splenectomy, *SPDP* spleen-preserving distal pancreatectomy, *PD* Pancreaticoduodenectomy, *MIPD* minimally invasive distal pancreatectomy, *BMI* body mass index, *ASA* American Society of Anesthesiologists

MIDP at high-volume centers was associated to lower postoperative mortality (0 vs 0.9%; 0.038), despite similar incidence of severe postoperative complications (11.6 vs 7.5%; *p* = 0.102) and longer operative time (256 [210–333] vs 240 [195–300]; *p* = 0.006). MIDP at high-volume centers was associated also to higher number of examined lymph nodes (26 [18–38] vs 15 [11–23]; *p* = 0.0001) but higher R1 rate (26.7 vs 8.3%; *p* = 0.004).

MIPD at high-volume centers was associated with lower intraoperative blood loss (200 [100–300] ml vs 300 [150–500] ml; *p* = 0.0001) and lower conversion to open surgery (4.9% vs 22.7%; *p* = 0.0001), despite older patient age, higher BMI, and higher proportion of ASA score ≥ 3.

## Discussion

This report of 1,191 contemporary MIPR from the prospective IGOMIPS registry provides some important information. First, robotic assistance was preferred for all types of MIPR but DPWS. Second, robotic assistance was associated with reduced rates of conversion to open surgery and lower amount of estimated blood loss. Third, robotic PD had trend toward lower mortality when compared to laparoscopic PD. Fourth, despite the use of robotic assistance was prevalent for SPDP, it did not increase the rate of spleen preservation. Fifth, DPWS was associated with higher conversion rate when compared to SPDP. Sixth, pancreatico-jejunostomy was prevalent in PD, but the technique of pancreatic anastomosis showed considerable variation. Seventh, performing ≥ 20 MIPR per year was associated with lower postoperative mortality and higher number of examined lymph nodes in DP and lower conversion to open surgery in SPDP. Performing ≥ 20 MIPD was associated with lower intraoperative blood loss and lower conversion to open surgery despite older patient age, higher BMI, and higher ASA score. Complex MIPR resections (i.e., PD and SPDP) were mostly performed at high-volume centers. Eight, most MIPR were performed by a single surgeon irrespective of center volume, but at high-volume centers, one-third of DP were performed by multiple surgeons as compared to < 20% for DPWS and < 5% for SPDP at low-volume centers. The importance of these findings is emphasized by the fact that figures refer to contemporary daily practice of MIPR on a national basis.

This registry analysis raises also important questions about the reliability of R1 assessment (i.e., the importance of standardized pathology of specimens) and the consequences of unplanned MIPR especially when this means an increase in technical complexity (e.g., from MIDP to MIPD).

Despite the lack of clear evidence of superiority of robotic assistance over the conventional laparoscopy in MIPR, the use of robotic assistance was prevalent for all types of MIPR but DPWS and increased over time. There has been a tenfold increase in robotic MIPR between 2019 and 2022, at hospitals where a robot is available. Recent evidence shows that robotic assistance outperforms laparoscopy in MIDP, for some outcome measures [17–19]. Considering the high costs of robotic assistance and the need to select the procedures in which this new technology may be conveniently employed, it is not surprising that the use of conventional laparoscopy was prevalent for DPWS. For MIPD, advantages of robotic assistance are more evident [20–22] as shown also by the high implementation of robotic PD in the IGOMIPS registry. One of the most striking pieces of evidence favoring robotic PD is provided by the Dutch trial on laparoscopic versus open PD that was terminated due to excess mortality in the laparoscopic arm [23]. Since then, Dutch surgeons have embraced robotic PD and have achieved excellent outcomes [24].

Little doubt exists that the use of robotic assistance reduces the rate of conversion to open surgery and the amount of blood loss [17–22]. The IGOMIPS registry confirms these results in daily practice, showing that advantages of robotic assistance in MIPR are not reserved to the few centers that have pioneered robotic surgery.

One striking result from this registry analysis is that robotic assistance, when compared to laparoscopy, reduced the incidence of severe postoperative complications, and could reduce postoperative mortality of MIPD. Despite higher prevalence of patients at increased operative risk (ASA score ≥ 3: 47.1% versus 36.8%; *p* = 0.045), incidence of severe postoperative complications was lower in robotic PD. Difference in mortality showed only a trend toward statistical significance. However, it may still be important to note that robotic PD was associated with a mortality rate of 3% at a national level. This mortality rate is equivalent to the value reported in the benchmark study for open PD when patients have an ASA score ≥ 3[25]. In robotic PD, approximately 50% of the patients had an ASA score ≥ 3.

Not surprisingly, MIPD performed at low-volume centers was associated with worse outcomes. Only 5 of 19 centers (26.3%) performing MIPD met the threshold of ≥ 20 procedures per year defined by the Miami guidelines [9]. Considering that approximately 30–40% of all PD can be MIPD, meeting this cut-off means that at least 50 PD are performed annually. Although just few centers met this annual volume at a national level, the importance of annual volume for the outcome of PD is well established [26]. A Dutch study showed that at least 40 PD per year are required to improve postoperative mortality [27]. A more recent study from Norway showed that ≥ 40 PD may not be enough to reduce postoperative mortality [28], and a study Korea demonstrated that mortality improves if the annual volume of PD is ≥ 54 [29]. Therefore, the annual volume ≥ 50 PD, permitting ≥ 20 MIPD, seems appropriate to offer good clinical outcomes.

This analysis also shows that most MIPR are performed by a single surgeon at most centers. This was especially true for MIPD. If, on the one hand, convincing evidence demonstrates that 250 robotic PD are required to achieve truly optimal outcomes [8], thus reinforcing the need for centralization, on the other hand, this high number of procedures raises the difficult question about how MIPD can safely diffuse on a large scale [30]. This study raises also important questions on how to train the next generation of pancreatic surgeons, and how to retrain the current generation of pancreatic surgeons that is mostly composed by open surgeons.

SPDP was mostly performed at high-volume centers, further underscoring the importance of volume in MIPR. Preserving the spleen is believed to be important in the rare patients with a benign but symptomatic tumor or a premalignant pancreatic tumor that require an MIDP. Spleen preservation prevents overwhelming sepsis and thrombocytosis and preserves overall immune function [31–33]. In addition, it could reduce blood loss and operative time, while limiting the rate of postoperative pancreatic fistula and delayed gastric emptying [31, 34–36]. However, SPDP, especially when the splenic vessels are also spared (Kimura procedure), is technically demanding and requires greater technical skills when compared to DPWS. This is mostly why robotic assistance is believed to improve the ability to preserve the spleen during MIDP [17, 18]. The fact that SPDP was mostly performed at high-volume centers is a possible explanation for the lack of an increased spleen preservation rate in the robotic group in this registry analysis.

In the IGOMIPS registry, DPWS was associated with higher rates of conversion to open surgery when compared to SPDP. In general, SPDP is technically more complex than DPWS. However, SPDP can be converted to DPWS when spleen preservation is not feasible, while primary DPWS is more frequently associated with difficulty factors, such as malignant histology, tumor proximity to the superior mesenteric-portal vein, sinistral portal hypertension, and splenomegaly making conversion to open surgery more likely to occur in these patients [37].

This registry analysis also showed that in MIPD, the pancreatic anastomosis is nearly always an end-to-side pancreatojejunostomy, but the surgical technique varied considerably among centers. A Blumgart or a modified Blumgart pancreatico-jejunostomy was used in 168 patients (38.6%), being the technique used more frequently. Practice in MIPD is probably influenced by experience in open PD. However, the minimally invasive approach may put additional difficulties, sometimes forcing surgeons to oversimplify the technique. This is probably why some surgeons prefer single layer running pancreatojejunostomy [38], despite this technique was associated with increased rates of postoperative pancreatic fistula in a large multicenter study [39]. The Blumgart technique is quite easy to perform during MIPD and combines the principle of duct-to-mucosa anastomosis to jejunal wrapping over the pancreatic stump. A recent study showed that modified Blumgart pancreatojejunostomy is associated with low rates of grade C postoperative pancreatic fistula in either open or robotic PD [40]. A modified Blumgart anastomosis is included in the standardized training pathway developed by the Dutch Pancreatic Cancer Group [24].

One key information from this study is that implementation of MIPR was not associated with high rates of resection for benign tumors, such as serous cystadenoma. A recent study showed that approximately 2% of the patients undergoing surgery for an incidentally discovered pancreatic cystic lesion have a final histology of serous cystadenoma [41]. Despite the different denominator in this study and in the IGOMIPS registry, the 3% rate of resection for serous cystadenoma reported herein is quite reassuring that availability of MI techniques does not result in unnecessary surgery [42].

This registry analysis showed conflicting data about R1 rates. DP at high-volume centers was associated with higher rates of R1 resection, which appears counterintuitive. Furthermore, robotic PD was associated with higher rates of R1 resection, while robotic DP showed lower R1 resection, versus laparoscopy. Margins status is an important quality metric in pancreatic surgery [43], but objective assessment relies on standardized and high quality of pathology. The higher number of examined lymph nodes suggests more accurate histology at high-volume centers, supporting the hypothesis of underestimation of R1 at low-volume centers. In addition, administration on neoadjuvant treatments decreases R1 rates [44], and a study from Esposito et al. showed that most resections for pancreatic cancer is R1 [45]. It is therefore difficult to believe that at high-volume centers, where patients receive neoadjuvant chemotherapy more frequently, R1 rates are truly higher when compared to low-volume centers. Clearly, quality of pathology makes most of the difference. Pathology of pancreatic specimens should become truly standardized to permit meaningful comparison on margin status.

This study has several limitations. First, despite prospective enrollment in the IGOMIPS registry, accuracy of information depends on individual centers. However, prospective data acquisition is the best possible method to ensure high quality of information. Second, some results may be influenced by local practice and/or quality of some hospital services (e.g., pathology). The large number of cases reported to the registry is expected to dilute the effect of these confounders. Third, relatively few centers provided most cases. Therefore, even in a national registry, quality of care mostly refers to specialized centers.

In conclusion, this registry analysis shows that MIPR can be safely implemented on a national scale. A few high-volume pancreatic centers perform most procedures, but results achieved at low-volume centers appears acceptable. Further analysis, on a larger sample, is required to understand nuances of implementation of MIPR in Italy.

### Supplementary Information

Below is the link to the electronic supplementary material.

Supplementary file1 (DOCX 14 KB)

## Data Availability

The datasets generated during the current study are available from the corresponding author on reasonable request. All materials are available upon request.
