# Correction to: Prospective minimally invasive pancreatic resections from the IGOMIPS registry: a snapshot of daily practice in Italy on 1191 between 2019 and 2022

**DOI:** 10.1007/s13304-023-01709-y

**Published:** 2023-12-02

**Authors:** Ugo Boggi, Greta Donisi, Niccolò Napoli, Stefano Partelli, Alessandro Esposito, Giovanni Ferrari, Giovanni Butturini, Luca Morelli, Mohammad Abu Hilal, Massimo Viola, Fabrizio Di Benedetto, Roberto Troisi, Marco Vivarelli, Elio Jovine, Alessandro Ferrero, Umberto Bracale, Sergio Alfieri, Riccardo Casadei, Giorgio Ercolani, Luca Moraldi, Carlo Molino, Raffaele Dalla Valle, Giuseppe Ettorre, Riccardo Memeo, Giacomo Zanus, Andrea Belli, Salvatore Gruttadauria, Alberto Brolese, Andrea Coratti, Gianluca Garulli, Renato Romagnoli, Marco Massani, Felice Borghi, Giulio Belli, Roberto Coppola, Massimo Falconi, Roberto Salvia, Alessandro Zerbi, the IGOMPIS registry, Emanuele F. Kauffmann, Giovanni Capretti, Luana Genova, Matteo De Pastena, Michele Mazzola, Alessandro Giardino, Matteo Palmieri, Alberto Manzoni, Vittoria Barbieri, Roberto Ballarin, Gianluca Rompianesi, Roberta Rossi, Laura Mastrangelo, Serena Langella, Mariangela Ilardi, Roberta Menghi, Claudio Ricci, Andrea Gardini, Donata Campra, Enrico Crolla, Sara Cecconi, Roberto L. Meniconi, Valentina Ferraro, Marco Brizzolari, Francesco Izzo, Davide Cintorino, Stefano Marcucci, Giuseppe Giuliani, Luigi Veneroni, Francesco Moro, Cristina Nistri, Damiano Caputo, Baiocchi Gianluca, Vincenzo Mazzaferro

**Affiliations:** 1 Division of General and Transplant Surgery, University of Pisa, Pisa, Italy; 2 Pancreatic Surgery Unit, Department of Biomedical Sciences, Humanitas University, Pieve Emanuele, Italy; 3 Pancreatic Surgery Unit, Pancreas Translational and Clinical Research Center, OSR ENETS Center of Excellence, IRCCS San Raffaele Scientific Institute, Milan, Italy; 4 General and Pancreatic Surgery Unit, Pancreas Institute, University of Verona, Verona, Italy; 5 Division of Minimally-Invasive Surgical Oncology, ASST Grande Ospedale Metropolitano Niguarda, Milan, Italy; 6 Department of Surgery, Pederzoli Hospital, Peschiera, Italy; 7 General Surgery, Department of Translational Research and New Technologies in Medicine and Surgery, University of Pisa, Pisa, Italy; 8 Department of Surgery, Poliambulanza Foundation Hospital, Brescia, Italy; 9 Department of Surgery, Ospedale Card. G. Panico, Tricase, Italy; 10 Hepato-Pancreato-Biliary Surgery and Liver Transplantation Unit, University of Modena and Reggio Emilia, Modena, Italy; 11 Division of HPB Minimally Invasive and Robotic Surgery, Department of Clinical Medicine and Surgery, Federico II University Hospital, Naples, Italy; 12 Hepatobiliary and Abdominal Transplantation Surgery, Department of Experimental and Clinical Medicine, Riuniti Hospital, Polytechnic University of Marche, Ancona, Italy; 13 Department of General Surgery, IRCCS, Azienda Ospedaliero-Universitaria di Bologna, Maggiore Hospital, Bologna, Italy; 14 Department of General and Oncological Surgery, “Umberto I” Mauriziano Hospital, Turin, Italy; 15 Department Clinical Medicine and Surgery, Federico II University of Naples, Via Pansini 5, 80131 Naples, Italy; 16 Digestive Surgery, Fondazione Policlinico Universitario A. Gemelli, IRCCS, Catholic University, Rome, Italy; 17 Division of Pancreatic Surgery, Department of Internal Medicine and Surgery (DIMEC), IRCCS, Azienda Ospedaliero Universitaria di Bologna Alma Mater Studiorum, University of Bologna, Bologna, Italy; 18 General and Oncology Surgery, Morgagni-Pierantoni Hospital, Forli, Italy; 19 Division of Oncologic Surgery and Robotics, Department of Oncology, Careggi University Hospital, Florence, Italy; 20 Department of Oncological Surgery Team 1, “Antonio Cardarelli” Hospital, Naples, Italy; 21 Hepatobiliary Surgery Unit Department of Medicine and Surgery, University of Parma, Parma, Italy; 22 Transplantation Department, S. Camillo-Forlanini Hospital, Rome, Italy; 23 Department of Hepato-Pancreatic-Biliary Surgery, General Regional Hospital “F. Miulli”, Acquaviva Delle Fonti, Bari, Italy; 24 4th Surgery Unit, Azienda ULSS2 Marca Trevigiana, Treviso, Italy; 25 Division of Hepatobiliary Surgical Oncology, Istituto Nazionale Tumori IRCCS Fondazione Pascale–IRCCS di Napoli, Naples, Italy; 26 Abdominal Surgery and Organ Transplantation Unit, ISMETT, Palermo, Italy; 27 Department of General Surgery and HPB Unit, Santa Chiara Hospital, Trento, Italy; 28 USL Toscana Sud Est, Misericordia Hospital, Grosseto, Italy; 29 General Surgery Unit, Infermi Hospital, Rimini, Italy; 30 Liver Transplant Center-General Surgery 2U, University of Turin, AOU Città della Salute e della Scienza di Torino, Turin, Italy; 31 Department of Surgery, Regional Hospital of Treviso, Treviso, Italy; 32 IRCCS Candiolo, Turin, Italy; 33 Ospedale Loreto Mare, Naples, Italy; 34 Department of Surgery, University Campus Bio-Medico of Rome, Fondazione Policlinico Universitario Campus Bio-Medico, Rome, Italy; 35 IRCCS Humanitas Research Hospital, Rozzano, Italy; 36 EndoCAS (Center for Computer Assisted Surgery), University of Pisa, Pisa, Italy; 37 Vita-Salute San Raffaele University, Milan, Italy

**Correction to: Updates in Surgery (2023) 75:1439–1456** 10.1007/s13304-023-01592-7

After publication online, the author name Under the IGOMPIS registry in html version was incorrectly published as Pastena Matteo.

The correct name is copied below

De Pastena Matteo

