# Supplementary material for: Prospective minimally invasive pancreatic resections from the IGOMIPS registry: a snapshot of daily practice in Italy on 1191 between 2019 and 2022
Source: Updates Surg. 2023 Jul 20;75(6):1439–56. doi: 10.1007/s13304-023-01592-7 (PMC10435655; doi:10.1007/s13304-023-01592-7)
Supplement: Supplementary file 1 — Supplementary file1 (DOCX 14 KB) [file 13304_2023_1592_MOESM1_ESM.docx]

**IGOMIPS registry**

The following surgeons contributed patients to the IGOMIPS registry, but do not qualify for full authorships. They should be listed as COLLABORATORS.

Emanuele F Kauffman^1^, Giovanni Capretti^2^, Luana Genova^3^, De Pastena Matteo^4^, Michele Mazzola^5^, Alessandro Giardino^6^, Matteo Palmieri^7^, Alberto Manzoni^8^, Vittoria Barbieri^9^, Roberto Ballarin^10^, Gianluca Rompianesi^11^, Roberta Rossi^12^, Laura Mastrangelo^13^, Serena Langella^14^, Mariangela Ilardi^15^, Roberta Menghi^16^, Claudio Ricci^17^, Andrea Gardini^18^, Donata Campra^19^, Enrico Crolla^20^, Sara Cecconi^21^, Roberto L Meniconi^22^, Valentina Ferraro^23^, Marco Brizzolari^24^, Francesco Izzo^25^, Davide Cintorino^26^, Stefano Marcucci^27^, Giuseppe Giuliani^28^, Luigi Veneroni^29^, Francesco Moro^30^, Cristina Nistri^31^, Damiano Caputo^32^, Baiocchi Gianluca^33^, Vincenzo Mazzaferro^34^

**Affiliations**

1 Division of General and Transplant Surgery, University of Pisa, Pisa, Italy

2 Pancreatic Surgery Unit, Department of Biomedical Sciences, Humanitas University, Pieve Emanuele, Italy and IRCCS Humanitas Research Hospital, Rozzano, Italy,

3 Pancreatic Surgery Unit, Pancreas Translational & Clinical Research Center, OSR ENETS Center of Excellence, IRCCS San Raffaele Scientific Institute, Milan, Italy; Vita-Salute San Raffaele University, Milan, Italy

4 General and Pancreatic Surgery Unit, Pancreas Institute, University of Verona, Verona, Italy

5 Division of Minimally-Invasive Surgical Oncology, ASST Grande Ospedale Metropolitano Niguarda, Milan, Italy

6 Department of Surgery, Pederzoli Hospital, Peschiera, Italy

7 General Surgery, Department of Translational Research and New Technologies in Medicine and Surgery, University of Pisa, Italy; EndoCAS (Center for Computer Assisted Surgery), University of Pisa, Italy.

8 Department of Surgery, Poliambulanza Foundation Hospital, Brescia, Italy

9 Department of Surgery, Ospedale Card. G. Panico, Tricase, Italy

10 Hepato-pancreato-biliary Surgery and Liver Transplantation Unit, University of Modena and Reggio Emilia, Modena, Italy

11 Division of HPB Minimally Invasive and Robotic Surgery, Department of Clinical Medicine and Surgery, Federico II University Hospital, Naples, Italy

12 Hepatobiliary and Abdominal Transplantation Surgery, Department of Experimental and Clinical Medicine, Riuniti Hospital, Polytechnic University of Marche, Ancona, Italy

13 Department of General Surgery, IRCCS, Azienda Ospedaliero-Universitaria di Bologna, Maggiore Hospital, Bologna, Italy

14 Department of General and Oncological Surgery, "Umberto I" Mauriziano Hospital, Turin, Italy

15 Department Clinical Medicine and Surgery, Federico II University of Naples, Via Pansini 5, 80131, Naples, Italy

16 Digestive Surgery, Fondazione Policlinico Universitario A. Gemelli, IRCCS, Catholic University, Rome, Italy

17 Division of Pancreatic Surgery, IRCCS, Azienda Ospedaliero Universitaria di Bologna^,^Department of Internal Medicine and Surgery (DIMEC), Alma Mater Studiorum, University of Bologna, Italy

18 General and Oncology Surgery, Morgagni-Pierantoni Hospital, Forli, Italy

19 General and Emergency Surgery, University of Turin, AOU Città della Salute e della Scienza di Torino, Turin, Italy

20 Department of Oncological Surgery Team 1, "Antonio Cardarelli" Hospital, Naples, Italy

21 Hepatobiliary Surgery Unit Department of Medicine and Surgery University of Parma Parma Italy

22 Transplantation Department, S. Camillo-Forlanini Hospital, Rome, Italy

23 Department of Hepato-Pancreatic-Biliary Surgery, General Regional Hospital "F. Miulli", Acquaviva delle Fonti, Bari, Italy

24 4th Surgery Unit, Azienda ULSS2 Marca Trevigiana, Treviso, Italy

25 Division of Hepatobiliary Surgical Oncology, Istituto Nazionale Tumori IRCCS Fondazione Pascale - IRCCS di Napoli, Naples, Italy

26 Abdominal Surgery and Organ Transplantation Unit, ISMETT, Palermo, Italy

27 Department of General Surgery and HPB Unit, Santa Chiara Hospital, Trento, Italy

28 USL Toscana Sud Est, Misericordia Hospital, Grosseto, Italy

29 General Surgery Unit, Infermi Hospital, Rimini, RN, Italy

30 Liver Transplant Center -General Surgery 2U, University of Turin, AOU Città della Salute e della Scienza di Torino, Turin, Italy

31 Department of Surgery, Regional Hospital of Treviso, Italy

32 Department of Surgery, University Campus Bio-Medico of Rome, Fondazione Policlinico Universitario Campus Bio-Medico, Rome, Italy

33 Ospedale Maggiore di Cremona, Cremona, Italy

34 Istituto Nazionale Tumori di Milano, Milano, Italy
